# Possibility of Controlling Self-Organized Patterns with Totalistic Cellular Automata Consisting of Both Rules like Game of Life and Rules Producing Turing Patterns

**DOI:** 10.3390/mi9070339

**Published:** 2018-07-03

**Authors:** Takeshi Ishida

**Affiliations:** Department of Ocean Mechanical Engineering, National Fisheries University, Shimonoseki 759-6595, Japan; ishida07@ecoinfo.jp; Tel.: +81-832-86-5111

**Keywords:** cellular automata, Game of Life, reaction-diffusion system, self-organization, Turing pattern model, Young model

## Abstract

The basic rules of self-organization using a totalistic cellular automaton (CA) were investigated, for which the cell state was determined by summing the states of neighboring cells, like in Conway’s Game of Life. This study used a short-range and long-range summation of the cell states around the focal cell. These resemble reaction-diffusion (RD) equations, in which self-organizing behavior emerges from interactions between an activating factor and an inhibiting factor. In addition, Game-of-Life-type rules, in which a cell cannot survive when adjoined by too many or too few living cells, were applied. Our model was able to mimic patterns characteristic of biological cells, including movement, growth, and reproduction. This result suggests the possibility of controlling self-organized patterns. Our model can also be applied to the control of engineering systems, such as multirobot swarms and self-assembling microrobots.

## 1. Introduction

Self-organization phenomena, in which global structures are produced from purely local interactions, are found in fields ranging from biology to human societies. If these emergent processes were to be controlled, various applications would be possible in engineering fields. For this purpose, the conditions under which they emerge must be elucidated. This is one of the goals of the study of complex systems. Such control methods may allow the automatic construction of machines. They could also be applied to the control of engineering systems, such as controlling robot swarms or self-assembling microrobots. In addition, the methods allow for the growth of artificial organs or the support of ecosystem conservation, as well as clarifying the emergence of the first life on ancient earth.

Mathematical modeling of self-organization phenomena has two main branches: the mathematical analysis of reaction-diffusion (RD) equations, and discrete modeling using cellular automata (CA).

The Turing pattern model is one type of RD model. This was introduced by Turing in 1952 [1], where he treated morphogenesis as the interaction between activating and inhibiting factors. Typically, this model achieves self-organization through the different diffusion coefficients for two morphogens, equivalent to an activating and an inhibiting factor. The general RD equations can be written as follows:

$$\frac{\partial u}{\partial t}=d_{1}\nabla^{2}u+f\left(u,v\right),$$

$$\frac{\partial v}{\partial t}=d_{2}\nabla^{2}v+g\left(u,v\right),$$

where *u* and *v* are the morphogen concentrations, functions f and g are the reaction kinetics, and $d_{1}$ and $d_{2}$ are the diffusion coefficients. Previous studies have considered a range of functions f and g, and models such as the linear model, the Gierer–Meinhardt model [2], and the Gray–Scott model [3] have been used to produce typical Turing patterns.

By contrast, CA models are discrete in both space and time. The state of the focal cell is determined by the states of the adjacent cells and the transition rules. The advantage of CA models is that they can describe systems that cannot be modeled using differential equations. Historically, various interesting CA patterns have been discovered.

In the 1950s, von Neumann introduced the mathematical study of self-reproduction phenomena, and proved, theoretically, that a self-replicating machine could be constructed on a two-dimensional (2D) grid using 29 cell states and transition rules [4]. Von Neumann’s self-reproducing machine was too large to be implemented until powerful computers became available in the 1990s [5]. Codd [6] showed that the number of cell states could be reduced to eight, and proved the possibility of a self-reproducing machine. However, this model was too complex to be applied to biological processes. In the 1970s, the Game of Life (GoL), invented by Conway [7], became popular. It is a simple CA model that is defined by only four rules, yet produces complex dynamic patterns. Wolfram [8] investigated the one-dimensional (1D) CA model and identified four categories of pattern formation. One of these was the chaotic behavior group. Langton [9] developed a simple self-reproducing machine that did not require von Neumann’s machine completeness. While having a very simple shape, Langton’s machine’s transition rules were complex and produced only specific circling shapes. Byl [10] simplified the Langton model, and Reggia [11] identified the simplest possible reproducing machine. Ishida [12] demonstrated a self-reproducing CA model that resembled a living cell, with DNA-like information carriers held inside a cell-like structure.

On the other hand, many studies have applied CA models to biological phenomena, such as the generation of seashell-like structures by Wolfram [8], the development by Young of an RD model in a CA setting that reproduced the patterns of animal coloration [13], the application of the Belousov–Zhabotinskii reaction CA model by Madore and Freeman [14], the catalytic modeling of proteins by Gerhardt and Schuster [15], the modeling of the immune system by De Boer and Hogeweg [16] and by Celada and Seiden [17], the tumor growth model of Moreira and Deutsch [18], the genetic disorder model of Moore and Hahn [19], the modeling of the hippocampus by Pytte et al. [20], and the dynamic modeling of cardiac conduction by Kaplan et al. [21]. Furthermore, Markus [22] demonstrated that a CA model could produce the same output as RD equations.

**Figure 1:**
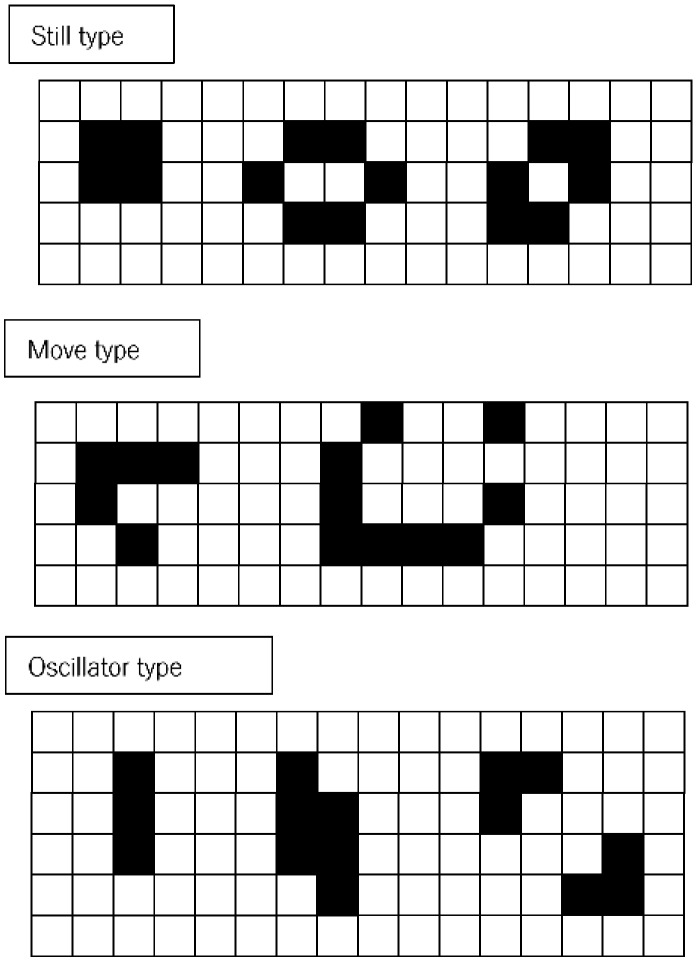
Patterns from Conway’s Game of Life under four rules: (1) a cell with one or no living neighbors dies; (2) a cell with four or more living neighbors dies; (3) a cell with three living neighbors is born; and (4) a cell with two or three living neighbors survives. The typical patterns that emerge can be categorized into three groups: still, moving, or oscillation.

In addition, CA has two types of model. The first one is a totalistic CA, for which each cell state is determined by summing the states of the neighboring cells. Conway’s GoL is one such CA. GoL produces the three typical patterns from the particular initial cell state configurations shown in Figure 1: “still”, “moving”, and “oscillating”. Meanwhile, most of the initial cell state configurations reach extinction, i.e., all cell states become 0. However, it is impossible to deduce the transition rules to make specific shapes automatically.

**Figure 2:**
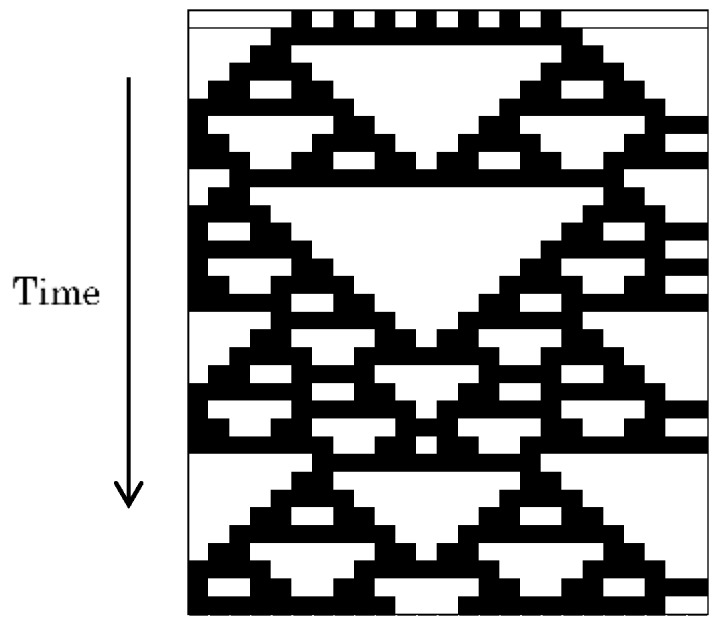
One of the patterns produced by Wolfram’s 1D cellular automaton (CA) model. Rule 126: white cell indicates the 0 state, while the black cell indicates the 1 state. The upper line corresponds to the initial condition. Applying this rule, the time series pattern produces a range of triangular shapes, in this case resembling a certain type of seashell.

In contrast with the totalistic CA, the second type of CA model counts all the neighbor cells’ state patterns. Wolfram’s 1D CA is one such CA, in which there are two states (0 and 1) of three inputs and one output. As there are 2^3^ = 8 inputs, and each input has two outputs (0 or 1), there are 2^8^ = 256 transition rules. Wolfram researched all these transition rules and divided the output patterns into four classes. Class 1 covers the steady states, Class 2 the periodical patterns, Class 3 the random patterns (edge of chaos), and Class 4 the chaotic patterns. Figure 2 shows one result of the Class 3 rules, which produce unique patterns like that of a certain type of seashell.

However, in a Wolfram-type model, increasing the inputs to five produces an explosive increase in the number of output combinations, to 2^32^. It therefore becomes impossible to investigate all outputs. A similar situation occurs in the case of a 2D CA model, making it, again, impossible to investigate all possible patterns. Langton proposed the λ parameter [23] to elucidate the condition to give rise to the diversity of patterns—edge of chaos. However, this could not derive the rule to construct favorite patterns.

CA models reduce the computational load by removing the need to solve differential equations numerically. However, it is necessary to translate the partial differential equations of the RD equations into the transition rules of the CA model. In a study of up to present, no general method currently exists for doing this, with the exception of the ultra-discrete systems [24] used in certain soliton equations. Normally, the transition rules driving the emergent phenomena must be found by trial and error. Studies that attempted to derive the transition rules using genetic algorithms [11,25] demonstrated the difficulty of deriving generalized rules. The Ishida cell division model [12] is a hybrid of the Wolfram- and Conway-type CAs, in which part of the rules are totalistic transition rules, and the others are head-to-head-type transition rules, and these rules were discovered by trial and error.

The Young model [13] is one of the 2D totalistic models that bridges the RD equations and CA model; this model is used to produce Turing patterns. Some other examples to produce Turing patterns are below. Adamatzky [26] studied a binary-cell-state eight-cell neighborhood two-dimensional cellular automaton model with semitotalistic transitions rules. Dormann [27] also used a 2D outer-totalistic model with threes states to produce a Turing-like pattern. Tsai [28] analyzed a self-replicating mechanism in Turing patterns with a minimal autocatalytic monomer-dimer system.

**Figure 3:**
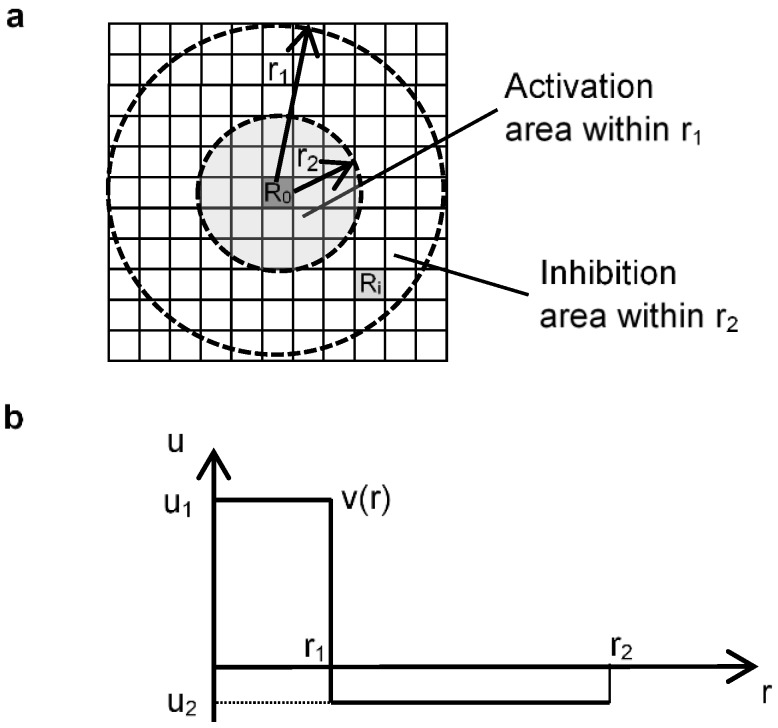
Outline of Young’s model. (**a**) The activation area has a radius r_1_ and the inhibition area has an outer radius r_2_; (**b**) Function v(r) is a continuous step function representing the activation area and inhibition area.

Young’s CA model uses a real number function to derive the distance effects, with two constant values within a grid cell: u_1_ (positive) and u_2_ (negative). The calculation begins by randomly distributing black cells on a rectangular grid. Then, for each cell at position R_0_ in 2D fields, the next cell state of R_0_, due to all nearby black cells at position R_i_, are added up. R_i_ is assumed to be a black cell within radius r_2_ from R_0_ cell. The function v(r) is a continuous step function, as shown in Figure 3b. The activation area, indicated by u_1_, has a radius of r_1_ and the inhibition area, indicated by u_2_, has a radius of r_2_ (r_2_ > r_1_). At position R_0_, when R_i_ is assumed to be a grid within r_2_, function v(|R_0_ − R_i_|) value is added up. Function |R_0_ − R_i_| indicates the distance between R_0_ and R_i_. If ∑_i_ v (|R_0_ − R_i_|) > 0, the grid cell at point R_0_ is marked as a black cell. If ∑_i_ v (|R_0_ − R_i_|) < 0, the grid cell becomes a white cell. If ∑_i_ v (|R_0_ − R_i_|) = 0, the grid cell does not change state [13]. Using these functions, Young described that a Turing pattern can be generated. Spot patterns or striped patterns can be created with relative changes between u_1_ and u_2_.

In this Young model, let u_1_ = 1, u_2_ = *w* (here 0 < *w* < 1), and further, if the state of the cell is set to 0 (white) and 1 (black), this model can be arranged as follows. The state of cell *i* is expressed as $c_{i}\left(t\right)$ ($c_{i}\left(t\right)$ = [0, 1]) at time *t*. The following state $c_{i}\left(t+1\right)$ at time t+1 is determined by the states of the neighboring cells. Here, *N*_1_ is the sum of the states of the domain within *S* meshes of the focal cell. Similarly, *N*_2_ is the sum of the states of the domain within *t* meshes of the focal cell, assuming that *S* < *t.*

$$N_{1}=\overset{S}{\underset{i=1}{\sum }}c_{i}\left(t\right)$$

$$N_{2}=\overset{T}{\underset{i=1}{\sum }}c_{i}\left(t\right)$$

**Figure 4:**
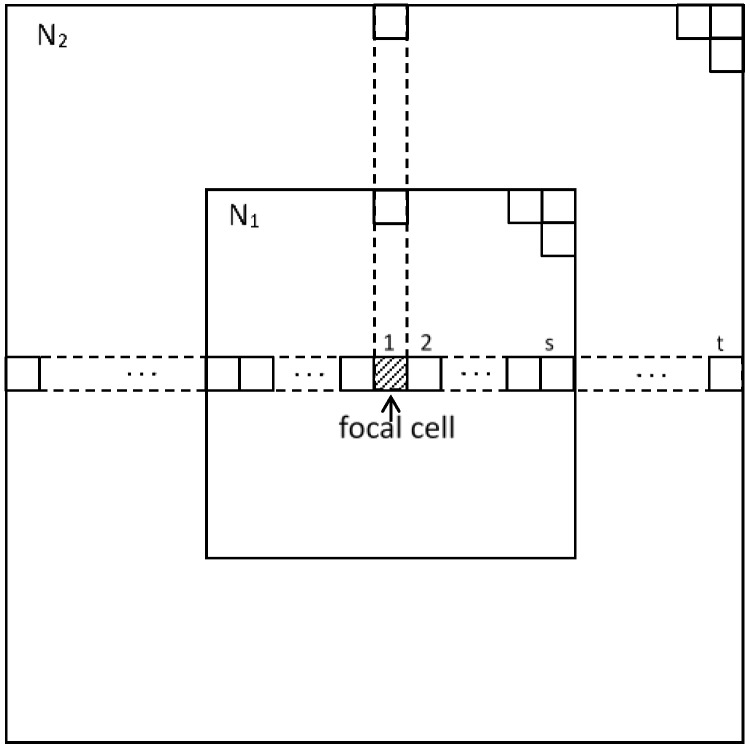
Schematic of the summing of states *N*_1_ and *N*_2_. Each grid cell has state 0 (white) or 1 (black). The inner area has a domain within *s* grids of the focal cell and the outer area a domain within *t* grids.

Here, *S (T)* is the number of cells within *s* (*t*) meshes from focal cell. Figure 4 shows the schematic of the total sum of states *N*_1_ and *N*_2_. The next time state of the focal cell is determined by the following Expression (1):

(1) $$\mathrm{Cell}\;\mathrm{state}\;\mathrm{at}\;\mathrm{the}\;\mathrm{next}\;\mathrm{time}\;\mathrm{step}\;=\{\begin{array}{c} 1:\;\mathrm{if}\;N_{1}>N_{2}\;\times \;w\\ \mathrm{Unchange}:\;\mathrm{if}\;N_{1}=N_{2}\;\times \;w\\ 0:\;\mathrm{if}\;N_{1}<N_{2}\;\times \;w \end{array}$$

The essence of the Turing pattern model is that the short- and long-range spatial scales are each affected by separate factors [29], and pattern formation emerges from nonlinear interactions between the two factors. Turing used two chemicals with different diffusion coefficients to create these short- and long-range spatial effects. However, as long as there exists a difference between long- and short-range effects, other implementation methods can be applied. This model used two ranges of *s* mesh and *t* mesh to create a difference. It is therefore thought that this model is essentially the same as an RD model. 

**Figure 5:**
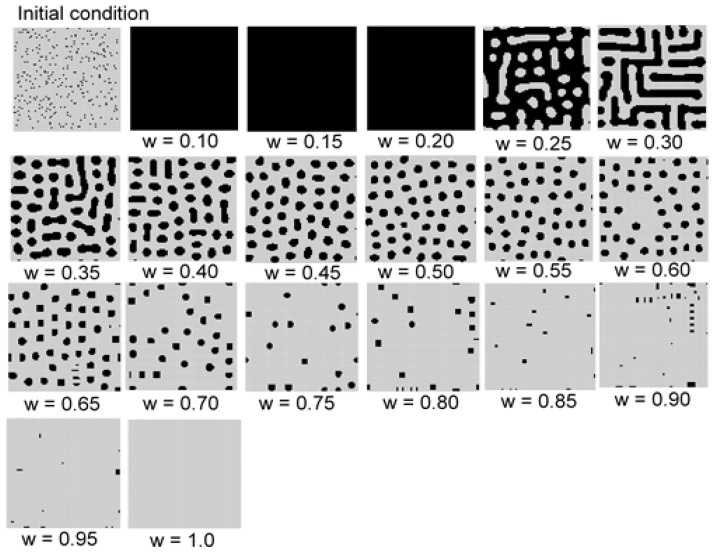
Results for the model of Expression (1) with *s* = 5 and *t* = 9 in a square grid. As the initial condition, 300 state 1 cells were set randomly in the calculation field. Turing-like patterns emerged as parameter *w* changed. When *w* was greater than 0.4, spot patterns were observed, whereas at *w* = 0.3, stripe patterns emerged. When *w* was 0.20 or lower, all cells in the field had a value of 1 (shown in black).

Figure 5 shows the results for the Expression (1) model using a square grid. As the initial conditions, 300 state 1 cells were set randomly in the calculation field 80 × 80. Turing-like patterns emerged as parameter *w* changed. When *w* was greater than 0.4, spot patterns were observed, whereas at *w* = 0.3, stripe patterns emerged. When *w* was 0.20 or lower, all cells in the field had a value of 1 (shown in black). Changing the parameters *N*_1_ and *N*_2_ merely changed the scale of the patterns.

On the other hand, the GoL is one of the 2D totalistic CAs to emerge patterns of diversity. In this model, the state of the focal cell is activated when the total number of surrounding states is within a certain range. Therefore, we considered the extended model of GoL. Whereas the GoL uses the states of the eight surrounding cells (the Moore neighborhood), an extended neighborhood is possible that considers two or more meshes from the focal cell like Young model. Such models are expected to generate a similar variety of patterns to the GoL.

In natural phenomena, short-distance influences play a stronger role than more distant influences. Under this circumstance (1) The influence decreases as the distance from the focal cell increases, which is *N*_1_ + *N*_2_ × *w* type model (*w*: weight parameter, 0 < *w* < 1). However, reaction diffusion phenomena are created with long-distance influences that play a negative role than more near influences. (2) The influence reverses as the distance from the focal cell increases, which is *N*_1_ *− N*_2_ *× w* type model (*w*: weight parameter, 0 < *w* < 1).

The first type of the model is expected to produce results similar to those of other totalistic CAs. Under specific conditions, GoL-like patterns will be found. The second model is expected to produce results similar to those of a Young model, which similarly takes account of the nearness of the activating and inhibiting factors.

This study adopted the second type of the model, and the emergence of a variety of patterns, such as the Turing pattern, were confirmed. In addition, by applying GoL-type rules to the second model type, more complex patterns are expected, such as self-reproducing and growth patterns. Our study model was able to produce not only a Turing-like pattern while using a simple transition expression, but also the model produces various patterns such as still, moving, and oscillation. Furthermore, this model can control these patterns with two parameters, which means the possibility of controlling self-organized patterns with a totalistic CA model.

## 2. Model

We investigated the results of an *N*_1_ − *N*_2_ × *w* type totalistic CA model in a 2D field that was implemented in Java. The GoL-like rules assumed in this model which limit the range of survival as state 1. To address this, by extending Expression (1), we introduced Expression (2). This expression was obtained by adding Rule 4 to Expression (1). When *N*_2_ = 0, it is assumed that Rule 4 takes precedence.

(2) $$\mathrm{Cell}\;\mathrm{state}\;\mathrm{of}\;\mathrm{the}\;\mathrm{next}\;\mathrm{time}\;\mathrm{step}\;=\{\begin{array}{c} 0:\;\mathrm{if}\;N_{1}>N_{2}\;\times \;w_{2}\;\left(Rule\;4\right)\\ 1:\;\mathrm{if}\;N_{1}>N_{2}\;\times \;w_{1}\;\left(Rule\;3\right)\\ \mathrm{Unchange}:\;\mathrm{if}\;N_{1}=N_{2}\;\times \;w_{1}\left(Rule\;2\right)\\ 0:\;\mathrm{if}\;N_{1}<N_{2}\;\times \;w_{1}\left(Rule\;1\right) \end{array}$$

$$N_{1}=\overset{S}{\underset{i=1}{\sum }}c_{i}\left(t\right)$$

$$N_{2}=\overset{T}{\underset{i=1}{\sum }}c_{i}\left(t\right)$$

Here, 1 > *w*_2_ > *w*_1_ > 0, and *N*_1_ is the sum of the states of the domain within *s* meshes of the focal cell. Similarly, *N*_2_ is the sum of the states of the domain within t meshes of the focal cell, assuming that *s* < *t*. *S* (*T*) is the number of cells within *s* (*t*) meshes from focal cell. As with the Young model, we used “Unchange” as the equality in Expression (2) to prevent all cells becoming state 1 or 0 in the next time step when *N*_1_ = 0 and *N*_2_ = 0.

**Figure 6:**
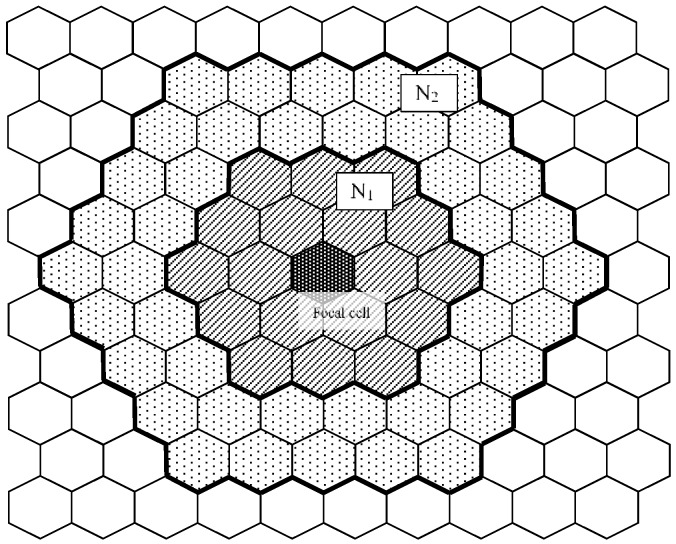
Hexagonal grid field. Here, *N*_1_ is the domain within s meshes of the focal cell. Similarly, *N*_2_ is the sum of the states of the domain within *t* meshes of the focal cell, assuming that *s* < *t*.

**Figure 7:**
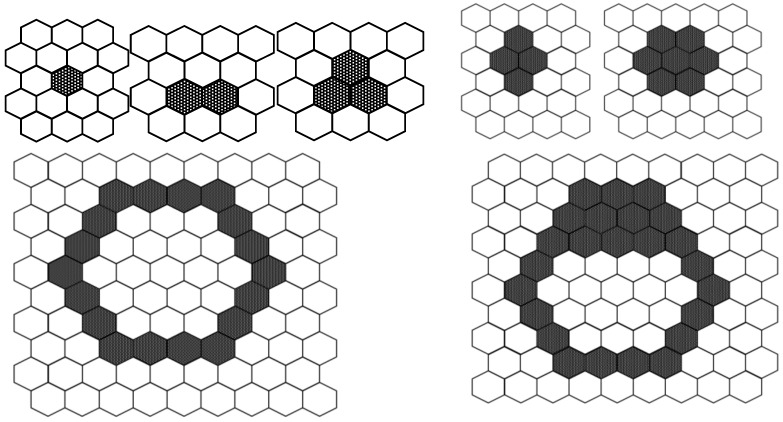
Initial conditions of hexagonal grids field. The black cell indicates the 1 state and the white cell indicates the 0 state. These configurations of state 1s were set in the center of the calculation fields.

The model used 2D hexagonal grids (Figure 6), in which the transition rules were simple to apply. Although square grids are generally used in 2D CA modeling, we also used hexagonal grids for the reason that a hexagonal grid is isotropic as opposed to a square grid. The models were implemented under the following conditions:

- Calculation field: 80 × 80 cells in hexagonal grids
- Periodic boundary condition
- Initial conditions were assumed following two types of conditions:
  Condition (a): some state 1s were set randomly in the calculation field;
  Condition (b): some state 1s were set in the center of the calculation fields (Figure 7).

## 3. Results

**Figure 8:**
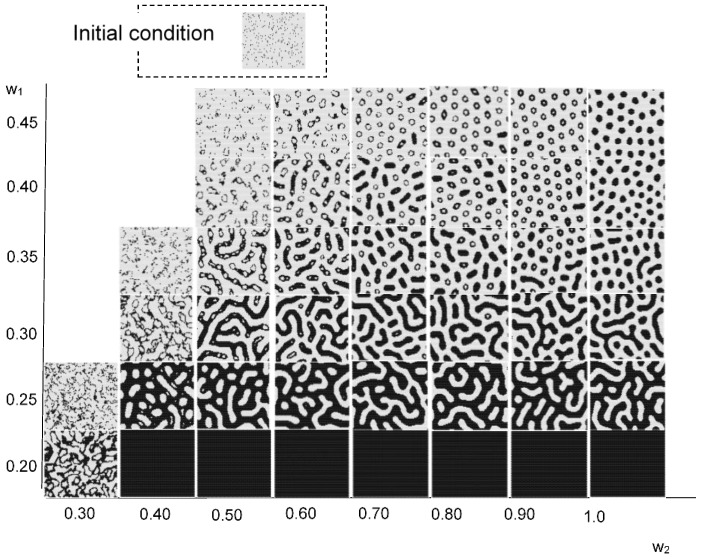
Results for the model with *s* = 5 and *t* = 9 in a hexagonal grid. The results image of each *w*_1_ and *w*_2_ value is based on 300 randomly arranged state 1 cells. Instability became stronger as *w*_2_ decreased to *w*_1_.

**Figure 9:**
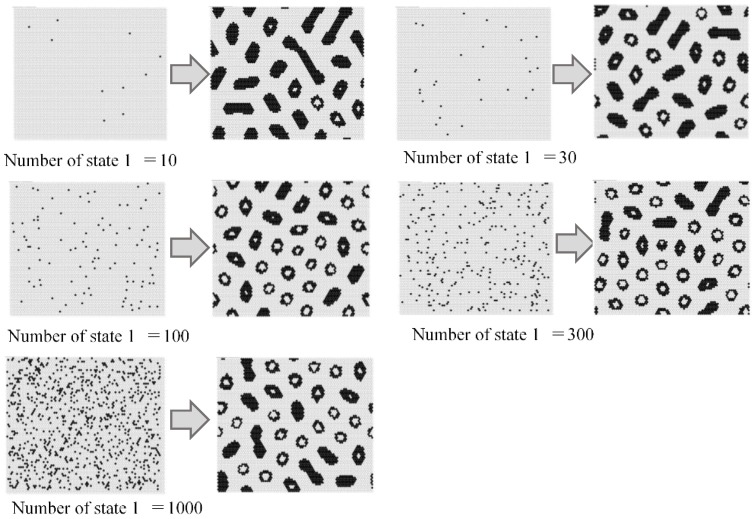
Results when varying the number of states 1 (black cell) to place them randomly in the initial states. The calculation conditions were *s* = 5, *t* = 9, *w*_1_ = 0.40, and *w*_2_ = 0.80 in a hexagonal grid. Based on this result, if there are more than a certain number of states 1, it is evident that a similar pattern formation develops.

**Figure 10:**
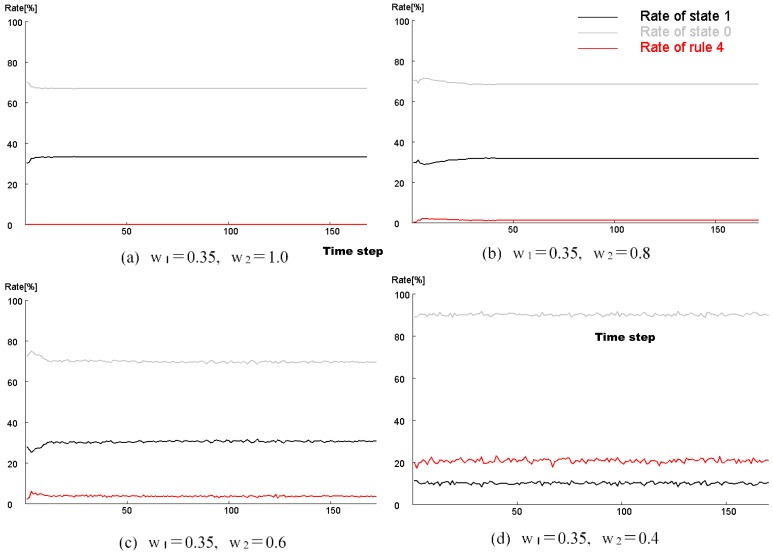
Transition ratio of states 1 and 2, and the ratio of the applied cells of Rule 4 at each time step. This result was calculated when *w*_2_ was changed in *s* = 5, *t* = 9, and *w*_1_ = 0.35. When *w*_2_ was 1, Rule 4 was not applied, and it was equivalent to the Young model. As *w*_2_ was lowered, the application ratio of Rule 4 increased. In comparison with Figure 8, white regions were generated in the black spots, and instability increased and began pulsating. This phenomenon is attributed to Rule 4.

Figure 8 maps the model results against parameters *w*_1_ and *w*_2_. Initial condition is based on 300 randomly arranged state 1 cells. Different patterns were observed at different values of *w*_1_ and *w*_2_. As in the Young-type model, the parameter *w*_1_ determined the pattern type, whether spotted or striped. As *w*_2_ became smaller, pulsing and unsteady white spots were born within the black pattern. The vibration of these white spots became more intense as the value of *w*_2_ was reduced. Figure 9 shows the results when varying the number of states 1 (black cell) to place them randomly in the initial state. The calculation conditions were *s* = 5, *t* = 9, *w*_1_ = 0.40, and *w*_2_ = 0.80 in a hexagonal grid. Based on the results of this figure, if there are more than a certain number of states 1, it is evident that a similar pattern formation develops. Furthermore, Figure 10 shows the transition of the ratio of states 1 and 2, and the ratio of the applied cells of Rule 4 at each time step. This result was calculated when *w*_2_ was changed (*s* = 5, *t* = 9, and *w*_1_ = 0.35). When *w*_2_ was 1, Rule 4 was not applied, and the result was equivalent to the Young model. As *w*_2_ was lowered, the application ratio of Rule 4 increased. At this time, white regions were generated in the black spots, and the instability increased and became pulsating. This phenomenon is attributed to Rule 4.

**Figure 11:**
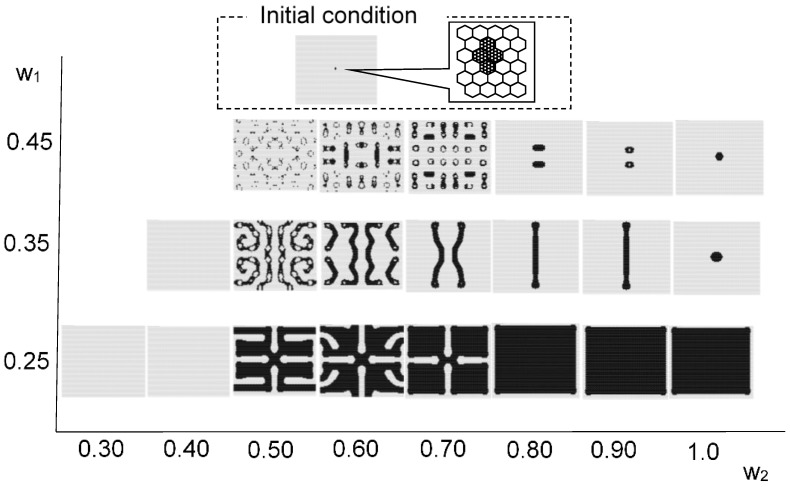
Results for the model with *s* = 5 and *t* = 9 in a hexagonal grid. The image of each w_1_ and *w*_2_ value is based on centrally arranged four black cells as shown in Figure 7. Instability became stronger as *w*_2_ decreased. The initial central spotted pattern grew as an unstable wave in the field. When *w*_2_ was relatively large, the central spot did not spread.

Figure 11 shows the effect of the initial condition which is centrally arranged on four black cells. The instability became more pronounced as *w*_2_ decreased, and the central spotted pattern grew as an unstable wave in the calculation field. By contrast, when *w*_2_ was relatively large, the central spot did not spread.

**Figure 12:**
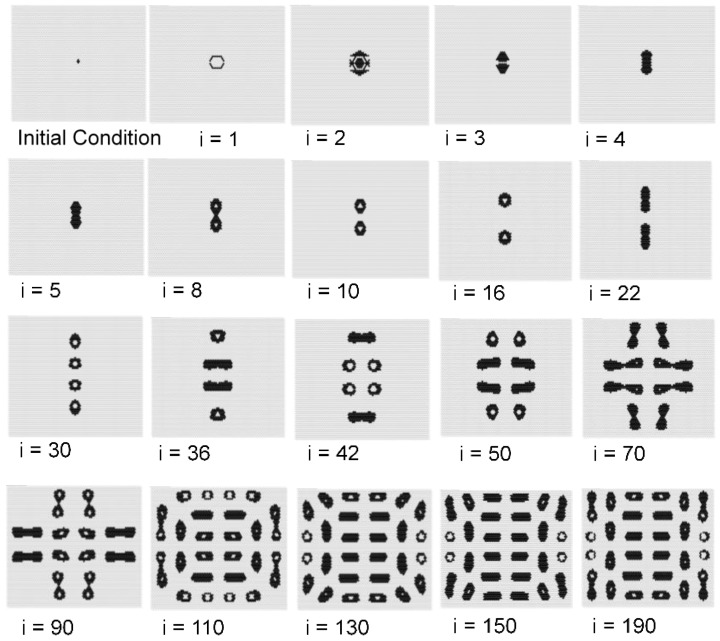
Results for the model with *s* = 4, *t* = 8, *w*_1_ = 0.40, and *w*_2_ = 0.75 in a hexagonal grid. As the initial condition, four state 1 cells (black) were set in the center of the field as shown in Figure 7. The central spotted pattern was divided into two spots, which then spread until the field was filled with spotted patterns.

**Figure 13:**
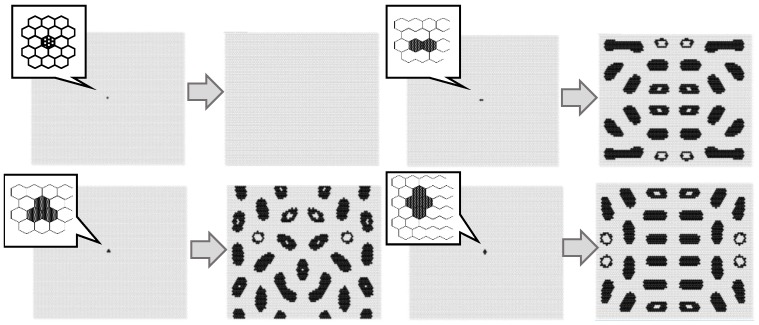
Results with the initial condition of state 1 under the same conditions as in Figure 12 (*s* = 4, *t* = 8, *w*_1_ = 0.40, and *w*_2_ = 0.75 in a hexagonal grid). If there is only one state 1 in the initial field, Rule 4 was applied at that location, which disappeared at the next step. If there are two or more state 1s as the initial state, state 1 was observed to survive, and a pattern formation similar to Figure 12 occurred.

Figure 12 shows the time series results of the model with *s* = 4, *t* = 8, *w*_1_ = 0.40, and *w*_2_ = 0.75. In this figure, *i* shows the iteration number of the calculation. The video of Figure 12 can be viewed in the Video S1 of Supplementary Materials. As the initial condition, four state-1 cells (black) were set in the center of the field shown in Figure 7. The central spotted pattern was divided into two spots, and these divisions spread until the field was filled with spotted patterns. Figure 13 shows the results when the initial condition of state 1 was subjected to the same conditions as in Figure 12 (*s* = 4, *t* = 8, *w*_1_ = 0.40, and *w*_2_ = 0.75 in a hexagonal grid). When there was only one state 1 in the initial field, Rule 4 was applied around this state 1, which disappeared at the next step. If there are two or more states 1 as the initial state, it was observed that the state 1 survived, and pattern formation similar to Figure 12 occurred. In the case of several state 1s, due to the balance between Rules 3 and 4, complicated patterns were observed in two or three steps from the beginning of the calculation, and thereafter, a self-replicating pattern was observed.

**Figure 14:**
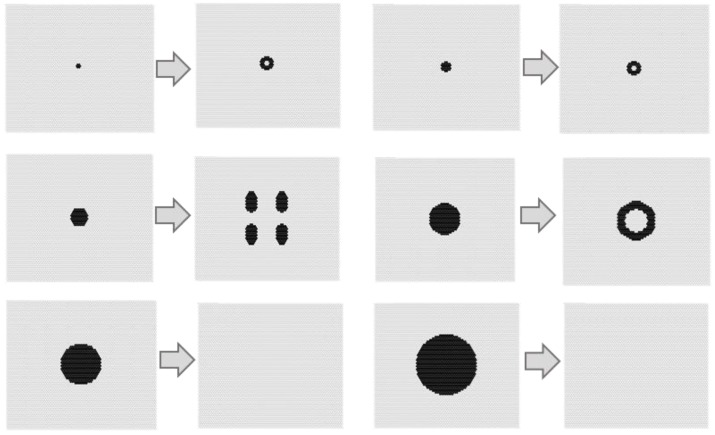
Calculation results, when increasing the black region as the initial condition, under the same conditions as in Figure 12 (*s* = 4, *t* = 8, *w*_1_ = 0.40, and *w*_2_ = 0.75 in a hexagonal grid). The point symmetrical initial shape tended to be a fixed pattern, due to the balance of each of the rules. On the other hand, if the black area is larger than a certain size, Rule 1 is mostly applied, and disappeared at the second step. To produce a self-replicating pattern, an asymmetric shape must initially occur.

**Figure 15:**
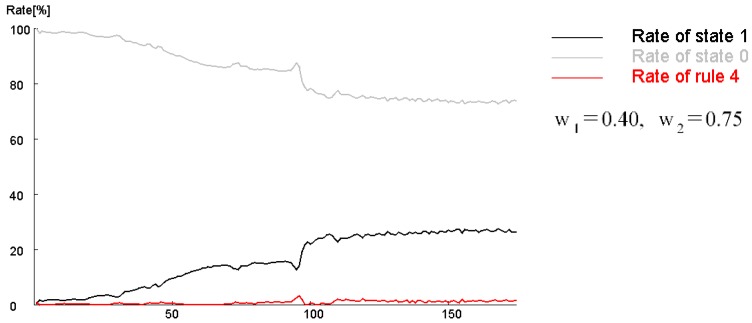
Transition of the ratios of states 1 and 2, and the ratio of the applied cells of Rule 4 per time step under the same conditions as in Figure 12 (*s* = 5, *t* = 9, *w*_1_ = 0.40, and *w*_2_ = 0.75). From 90 to 100 steps, the frequency of pattern division became high, and the application ratio of Rule 4 increased.

In addition, Figure 14 shows the calculation results when increasing the black region as the initial condition, under the same conditions as in Figure 12 (*s* = 4, *t* = 8, *w*_1_ = 0.40, and *w*_2_ = 0.75 in a hexagonal grid). The point symmetrical initial shape tended to be a fixed pattern due to the balance of the rules. On the other hand, if the black area was larger than a certain size, Rule 1 was mostly applied, and disappeared at the second step. To produce a self-replicating pattern, an asymmetric shape must initially occur. Figure 15 shows the transition of the ratios of states 1 and 2, and the ratio of applied cells of Rule 4 per time step under the same conditions as in Figure 12 (*s* = 5, *t* = 9, *w*_1_ = 0.40, and *w*_2_ = 0.75). In 90 to 100 steps, the frequency of pattern division became high, and the application ratio of Rule 4 increased. Rule 4 generated a white area in the black spot patterns, and instability increased; hence, the symmetrical shape collapsed and became a trigger for division.

**Figure 16:**
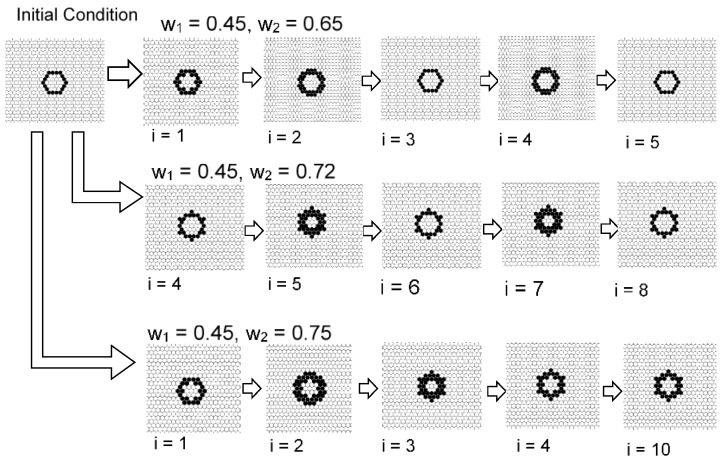
Results for the model with *s* = 5 and *t* = 8 in a hexagonal grid. The initial pattern was a symmetrical ring. When *w*_1_ = 0.45 and *w*_2_ = 0.65, the ring pattern became an oscillation pattern with period 2. When *w*_1_ = 0.45 and *w*_2_ = 0.72, the ring pattern likewise became an oscillation pattern with period 2. When *w*_1_= 0.45 and *w*_2_ = 0.75, a still pattern was formed.

**Figure 17:**
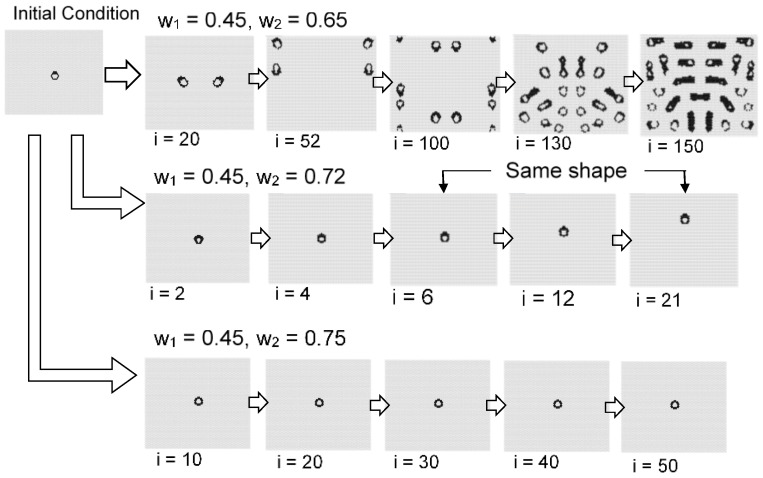
Results for the model with *s* = 5 and *t* = 8 in a hexagonal grid. The initial pattern was a symmetrical ring. When *w*_1_ = 0.45 and *w*_2_ = 0.48, the ring pattern disappeared. When *w*_1_ = 0.45 and *w*_2_ = 0.55, the ring pattern spread while breaking apart. When *w*_1_ = 0.45 and *w*_2_ = 0.60, still patterns were formed.

Figure 16 and Figure 17 show the results with an initial pattern of a symmetrical ring with state 1 s. In the case where the initial shape was symmetrical, the still pattern or the oscillation pattern often appeared in a wide range of *w*_1_ and *w*_2_. When *w*_1_ = 0.45 and *w*_2_ = 0.55, the ring pattern spread while dividing spots. In an opposite way, as *w*_2_ was approached as *w*_1_, the instability increased and the pattern disappeared.

**Figure 18:**
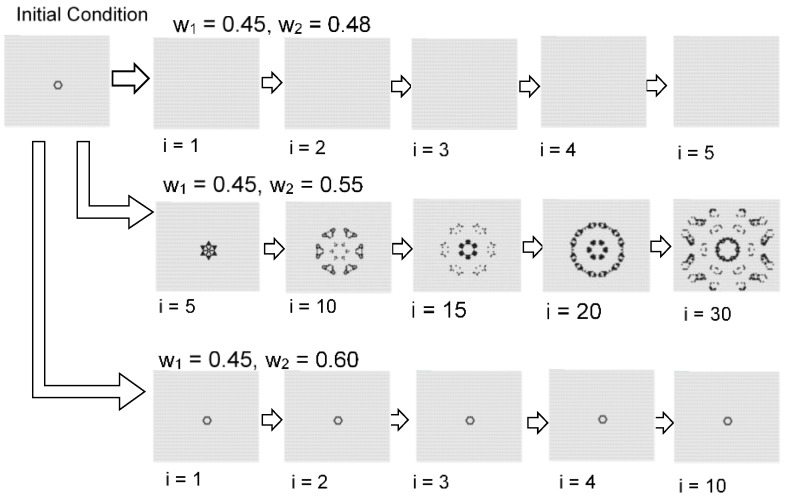
Results for the model with *s* = 5 and *t* = 8 in a hexagonal grid. The initial pattern was a deformed ring with a thick upper boundary. When *w*_1_ = 0.45 and *w*_2_ = 0.65, the ring pattern was divided while moving. When *w*_1_ = 0.45 and *w*_2_ = 0.72, a moving ring-like pattern formed. When *w*_1_ = 0.45 and *w*_2_ = 0.75, a single fixed symmetrical ring pattern formed.

Figure 18 shows the initial pattern of a deformed ring with a thick upper boundary. When *w*_1_ = 0.45 and *w*_2_ = 0.65, the ring pattern was divided while moving. When *w*_1_ = 0.45 and *w*_2_ = 0.72, a constantly moving ring-like pattern formed. When *w*_1_ = 0.45 and *w*_2_ = 0.75, a single fixed symmetrical ring pattern formed. Thus, when the initial shape is asymmetric, a moving pattern or dividing pattern tends to be generated from the breaking of symmetry. As *w*_2_ approaches 1, it approaches fixed patterns, and results in a pattern close to the Turing pattern.

## 4. Discussion

In this study, we constructed a model in which the focal state 1 survives only when a moderate rate of the number of state 1 cells surrounds the focal cell. This model created a central white part (state 0) within the black domain by changing parameter *w*_2_. The patterns then became increasingly unstable over the calculation field, and this instability increased the rate of pattern emergence, including self-reproducing spot patterns and stripe patterns.

The self-reproducing spot pattern of Figure 12 seems to have some association with the Gray–Scott model, which is a type of RD model. Generally, the Gray–Scott model produces a self-reproducing spot pattern when the parameters are adjusted. In future work, we will investigate the relationship between the current model and the Gray–Scott model.

Particularly, in the case of the model with *s* = 5 and *t* = 8 shown in Figure 16, Figure 17 and Figure 18, the result suggests the possibility of controlling self-organized patterns. When *w*_1_ is increased, a spot shape can be generated, and by decreasing *w*_1_, a stripe pattern can be grown from spotted seeds.

**Figure 19:**
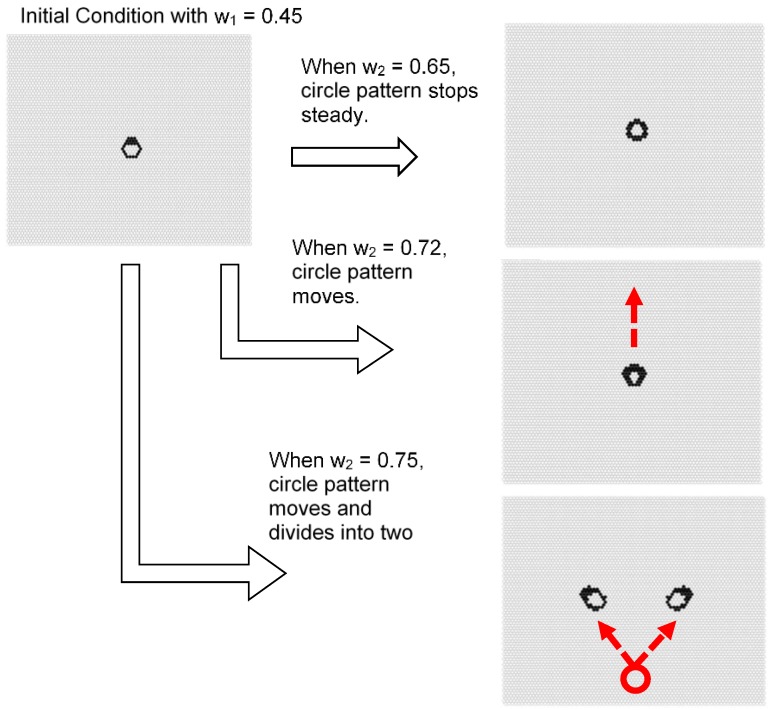
Possibility of self-organized ring patterns with one parameter. The results are a part of the model with *s* = 5 and *s* = 8 in a hexagonal grid, as shown in Figure 13. The ring pattern can be controlled with parameter *w*_2_. When *w*_2_ = 0.65, the ring pattern was divided while moving. When *w*_2_ = 0.72, a moving ring pattern formed. When *w*_2_ = 0.75, a fixed ring pattern formed.

In the symmetric shape, when *w*_2_ is large, it is stable in many cases. However, by making the value of *w*_2_ small and making it close to *w*_1_, the spotted pattern becomes unstable, and asymmetric shapes are born. Furthermore, these spots are moving or divided by the controlling *w*_2_ parameter, as shown in Figure 19. 

The robustness of the initial conditions was found to have the following tendencies. (1) If state 1 is one, it disappears; (2) When state 1 is randomly arranged, if two or more states 1 are distributed in the range for counting the number of surrounding states, the development of the pattern is recognized. Next, depending on the degree of instability due to the *w*_2_ parameter, it is decided whether the pattern develops spatially. Due to random initial placement, an identical pattern does not occur, but qualitatively similar patterns can be stably generated; (3) When several states 1 are gathered, complicated pattern changes occur in the first two or three steps due to the balance between Rules 3 and 4, and if a shape that is not point symmetric is subsequently generated, a pattern spreading in the space is generated. When a point symmetric shape is born, a circular or ring-fixed pattern is generated; (4) When the black (state 1) area is large, the black area disappears due to Rule 1.

In a future study, if CA rules can translate to a chemical reaction process, a control technology for nanomachines becomes feasible. Thus, the simple totalistic CA presented in this paper allows the emergence of a wide range of self-organization patterns to be observed.

**Figure 20:**
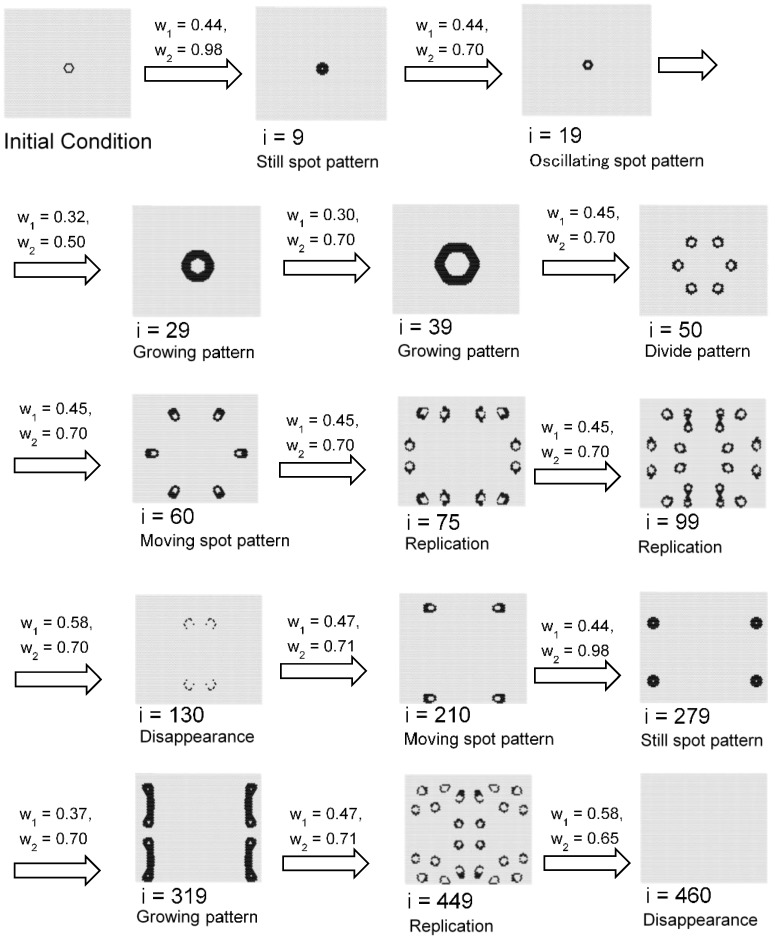
Calculation results in which *w*_1_ and *w*_2_ are changed over time at each step. The initial pattern was a hexagonal ring shape of state 1s, with *s* = 5 and *t* = 8 in a hexagonal grid. As in this result, it is possible to create changes by changing *w*_1_ and *w*_2_; when *i* = 1–9, *w*_1_ = 0.44, and *w*_2_ = 0.98, birth of annulus was observed; when *i* = 11–19, *w*_1_ = 0.44, and *w*_2_ = 0.70, an oscillating spot pattern was observed; when *i* = 20–29, *w*_1_ = 0.32, and *w*_2_ = 0.50, a growing ring pattern was observed; when *i* = 30–39, *w*_1_ = 0.30, and *w*_2_ = 0.70, a growing ring pattern was observed; when *i* = 40–99, *w*_1_ = 0.45, and *w*_2_ = 0.70, divide patterns and replication were observed; when *i* = 100–139, *w*_1_ = 0.58, and *w*_2_ = 0.70, pattern disappearance was observed; when *i* = 140–259, *w*_1_ = 0.47, and *w*_2_ = 0.71, moving spotted patterns were observed; when *i* = 260–279, *w*_1_ = 0.44, and *w*_2_ = 0.98, still circle patterns were observed; when *i* = 280–319, *w*_1_ = 0.37, and *w*_2_ = 0.70, a growing ring pattern was observed; when *i* = 320–449, *w*_1_ = 0.47, and *w*_2_ = 0.71, divide patterns and replication were observed; when *i* = 460–, *w*_1_ = 0.58, and *w*_2_ = 0.65, disappearance of all patterns was observed.

Based on these results, if the parameters *w*_1_ and *w*_2_ are changed at each time step, it is possible to generate a continuously changing pattern. Figure 20 shows a calculation case in which *w*_1_ and *w*_2_ are changed in time over time. The video of Figure 20 can be viewed in the Video S2 of Supplementary Materials. As in this result, it is possible to create changes by changing *w*_1_ and *w*_2_; birth of annulus -> growth -> division -> move -> decline -> growth -> annihilation.

**Figure 21:**
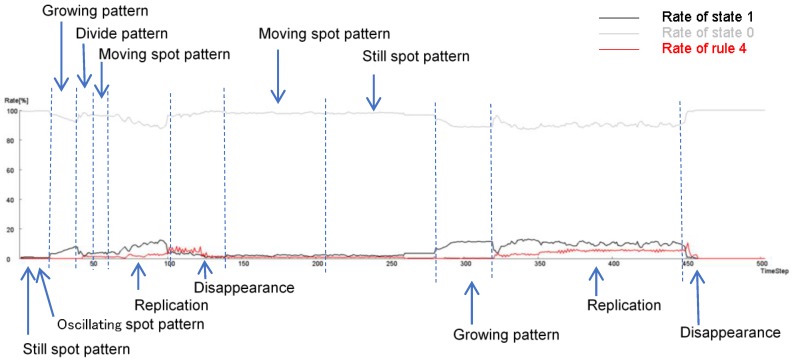
Transition of the ratio of states 1 and 2, and the ratio of the applied cells of Rule 4 in each time step during the calculation of Figure 20. Rule 4 was applied more frequently when spot patterns replicated than when the spot moved or vibrated. Furthermore, when the pattern disappeared, the application frequency of Rule 4 increased. Thus, it is suggested that pattern stability can be controlled by changing the application ratio of Rule 4.

Figure 21 shows the transition of the ratio of states 1 and 2, and the ratio of the applied cells of Rule 4 in each time step during the calculation of Figure 20. Rule 4 was applied more frequently when spot patterns replicated, as compared to when the spot moved or vibrated. Furthermore, when the pattern disappeared, the application frequency of Rule 4 increased. Thus, it is suggested that pattern stability can be controlled by changing the application ratio of Rule 4.

Since this research is still in its first stage, discussions are limited mainly to qualitative considerations, but in future work, it will be necessary to investigate further quantitative assessments. There is a possibility of finding an index like Langton’s λ parameter with the evaluation of many calculation cases. Furthermore, since it has rules similar to the Game of Life, basically like the Game of Life, many trials are necessary to find the desired pattern. Slight changes in the initial value may develop into different patterns. In the future, it is necessary to make a catalog of pattern formation on the relation between the initial condition and patterns based on many calculation results.

## 5. Conclusions

To observe special patterns of emergent behavior that mimic biological systems, we developed a totalistic CA model with an activating inner area and inhibiting outer area. This is a CA model, yet it works in a way that is equivalent to that of RD approaches like the Turing pattern model. If a multiple CA model can be designed that allows the number of states to be increased, we may witness the emergence of more complex patterns, like those of living things.

Our model also has potential applications in the engineering field. For example, it may be possible to develop swarming robots that can work as a single unit or divide into groups. As shown in the result of Figure 13, spot-like regions can be formed in the space by this algorithm. If many robots are dispersedly arranged in this space, it becomes possible to gather by grouping the robots within this spot-like rounded area. Furthermore, using this algorithm, it is also possible to divide one spot area into two. Therefore, robots can be divided into two groups simply by paying attention so that the robot that was in the first spot area does not come out of the area.

The model can further be applied to information networks. If the transition rules can be applied to Internet of Things (IoT) devices through the Web, hierarchical structures of IoT devices may emerge. For example, if the IoT devices know their own position, this algorithm can be applied by transmitting tokens to the nearby devices. Then, the spotted structure can be formed in the network space of devices, and the devices can be divided into plural groups. Furthermore, if our method can be applied to microsystems, it may become possible to make a production process of self-organizing a microrobot from nanoparts automatically.

The model also produces Turing-like patterns using a simple algorithm and without the need to solve complex simultaneous differential equations. However, a weak point is the necessity to add up the state quantities within a certain area. While these calculations are simple to perform using electronic devices, it is difficult to see how an individual living cell in an organism would be able to calculate the states of distant cells. To apply this model to morphogenesis, therefore, further modification is needed.

Other outstanding questions include the mathematical relationship between this model and the RD equations, and the relationship with a Turing machine. It would also be interesting to consider the entropy of the model.

## Supplementary Materials

The following are available online at http://www.mdpi.com/2072-666X/9/7/339/s1, Video S1: Video of Figure 12 (Results for the model with *s* = 4, *t* = 8, *w*_1_ = 0.40, and *w*_2_ = 0.75 in a hexagonal grid); Video S2: Video of Figure 20 (Calculation results in which *w*_1_ and *w*_2_ are changed over time at each step).

## References

[1] Turing A.M. (1952). The Chemical Basis of Morphogenesis. Philos. Trans. R. Soc. Lond. B Biol. Sci..

[2] Gierer A., Meinhardt H.A. (1972). A Theory of Biological Pattern Formation. Kybern. Biol. Cybern..

[3] Gray P., Scott S. (1984). Autocatalytic Reactions in the Isothermal Continuous Stirred Tank Reactor. Chem. Eng. Sci..

[4] Neumann J. (1966). Theory of Self-Replicating Automata.

[5] Mange D., Stauffer A., Peparodo L., Tempesti G.G. (2004). A Macroscopic View of Self-Replication. Proc. IEEE.

[6] Codd E.F. (1968). Cellular Automata.

[7] Gardner M. (1970). Mathematical Games–The fantastic combinations of John Conway’s new solitaire game “life”. Sci. Am..

[8] Wolfram S. (1984). Universality and Complexity in Cellular Automata. Physics D.

[9] Langton C. (1989). Artificial Life.

[10] Byl J. (1989). Self-Reproduction in Small Cellular Automata. Physics D.

[11] Reggia J.A., Lohn J.D., Chou H.H. (1998). Self-Replicating Structures: Evolution, Emergence, and Computation. Artif. Life.

[12] Ishida T. (2010). Simulating Self-reproduction of Cells in a Two-dimensional Cellular Automaton. J. Robot Mechatron..

[13] Young D.A.A. (1984). Local Activator-Inhibitor Model of Vertebrate Skin Patterns. Math. Biosci..

[14] Madore B.F., Freedman W.L. (1983). Computer Simulation of the Belousov-Zhabotinsky reaction. Science.

[15] Gerhardt M., Schuster H. (1989). A Cellular Automaton Describing the Formation of Spatially Ordered Structures in Chemical Systems. Phys. D.

[16] De Boer R.J., Hogeweg P., Perelson A.S. (1992). Growth and Recruitment in the Immune Network. Theoretical and Experimental Insights into Immunology.

[17] Celadaa F., Seidenb P.E. (1992). A Computer Model of Cellular Interactions in the Immune System. Immunol. Today.

[18] Moreira J., Deutsch A. (2002). Cellular Automaton Models of Tumor Development: A Critical Review. Adv. Complex Syst..

[19] Moore J.H., Hahn L.W. (2000). A Cellular Automata-based Pattern Recognition Approach to Identifying Gene-gene and Gene-environment Interactions. Am. J. Hum. Genet..

[20] Pytte E., Grinstein G., Traub R.D. (1991). Cellular Automaton Models of the CA3 Region of the Hippocampus. Network.

[21] Kaplan D.T., Smith J.M., Saxberg B.E.H., Cohen R.J. (1988). Nonlinear Dynamics in Cardiac Conduction. Math. Biosci..

[22] Markus M., Hess B. (1990). Isotropic Cellular Automaton for Modeling Excitable Media. Nature.

[23] Langton C.G. (1990). Computation at the edge of chaos: Phase transitions and emergent computation. Physics D.

[24] Tokihiro T., Takahashi D., Matsukidaira J., Satsuma J. (1996). From Soliton Equations to Integrable Cellular Automata through a Limiting Procedure. Phys. Rev. Lett..

[25] Sipper M. (1998). Fifty years on Self-Replicating; An Overview. Artif. Life.

[26] Adamatzky A., Martínez G.J., Carlos J., Mora S.T. (2006). Phenomenology of reaction-diffusion binary-state cellular automata. Int. J. Bifurc. Chaos.

[27] Dormann S., Deutsch A., Lawniczak A.T. (2001). Fourier analysis of Turing-like pattern formation in cellular automaton models. Future Gener. Comput. Syst..

[28] Tsai L.L., Hutchison G.R., Peacock-Lopez E. (2000). Turing patterns in a self-replicating mechanism with a self-complementary template. J. Chem. Phys..

[29] Nakamasu A., Takahashi M., Kondo S. (2009). Interactions between Zebrafish Pigment Cells Responsible for the Generation of Turing Patterns. Proc. Natl. Acad. Sci. USA.

